# Recognition of Leaf Disease Using Hybrid Convolutional Neural Network by Applying Feature Reduction

**DOI:** 10.3390/s22020575

**Published:** 2022-01-12

**Authors:** Prabhjot Kaur, Shilpi Harnal, Rajeev Tiwari, Shuchi Upadhyay, Surbhi Bhatia, Arwa Mashat, Aliaa M. Alabdali

**Affiliations:** 1 Chitkara University Institute of Engineering and Technology, Chitkara University, Rajpura 140401, Punjab, India; prabhjot.k@chitkara.edu.in (P.K.); shilpi13n@gmail.com (S.H.); 2 Department of Systemics, School of Computer Science, University of Petroleum and Energy Studies, Bidholi, Dehradun 248007, Uttarakhand, India; 3 Department of Allied Health Sciences, School of Health Sciences, University of Petroleum and Energy Studies, Bidholi, Dehradun 248007, Uttarakhand, India; shuchi.upadhyay@ddn.upes.ac.in; 4 Department of Information Systems, College of Computer Science and Information Technology, King Faisal University, P.O. Box 400, Hofuf 31982, Saudi Arabia; sbhatia@kfu.edu.sa; 5 Faculty of Computing and Information Technology, King Abdulaziz University, P.O. Box 344, Rabigh 21911, Saudi Arabia; aasmashat@kau.edu.sa (A.M.); amalabdali@kau.edu.sa (A.M.A.)

**Keywords:** convolutional neural network, image classification, transfer learning, EfficientNet B7, leaf disease detection, plant disease, feature reduction and extraction

## Abstract

Agriculture is crucial to the economic prosperity and development of India. Plant diseases can have a devastating influence towards food safety and a considerable loss in the production of agricultural products. Disease identification on the plant is essential for long-term agriculture sustainability. Manually monitoring plant diseases is difficult due to time limitations and the diversity of diseases. In the realm of agricultural inputs, automatic characterization of plant diseases is widely required. Based on performance out of all image-processing methods, is better suited for solving this task. This work investigates plant diseases in grapevines. Leaf blight, Black rot, stable, and Black measles are the four types of diseases found in grape plants. Several earlier research proposals using machine learning algorithms were created to detect one or two diseases in grape plant leaves; no one offers a complete detection of all four diseases. The photos are taken from the plant village dataset in order to use transfer learning to retrain the EfficientNet B7 deep architecture. Following the transfer learning, the collected features are down-sampled using a Logistic Regression technique. Finally, the most discriminant traits are identified with the highest constant accuracy of 98.7% using state-of-the-art classifiers after 92 epochs. Based on the simulation findings, an appropriate classifier for this application is also suggested. The proposed technique’s effectiveness is confirmed by a fair comparison to existing procedures.

## 1. Introduction

The agricultural sector is a significant and new framework for researchers in the field of computer vision today. Agriculture’s primary goal is to produce a wide range of valuable and substantial crops and plants. In farming, plant pathogens diminish the amount and characteristics of the product, so they must be controlled early [1]. Recently, agricultural researchers have focused their efforts on diseases of various fruits and crops. The researchers devised several methods for detecting and classifying diseases in fruits and crops [2,3]. Grapes seem to be a complex fruit to grow because the plants are constantly attacked by viruses, resulting in a significant reduction in grape production [4]. As a result, it is critical to control contaminated crops before they wreak havoc on product quality and quantity. Human inspections are mostly used for disease diagnosis, but some disadvantages make this procedure complicated, including time, expense, availability, and the need for many efforts. Various bacterial and fungal diseases manifest themselves primarily occur on the surface of leaf area and fruit area. Lesions, like pests, have complicated patterns that make them difficult to understand. Bacteria, on the other hand, thrive as single cells with a simpler life cycle. They reproduce by dividing a single cell into two; a method called binary fusion. Viruses are microscopic particles that contain genes and fibers but no membrane proteins [5]. On grape leaves, diseases like rust, scab, downy mildew with powdery mildew, and some more diseases like leaf blight, black measles, and black rot can occur. Image processing-based automated systems have recently been developed, which can efficiently identify and recognize diseases in Horticulture. The researchers use image processing to determine the diseased part’s location, color, form, scale, and boundaries. Pre-processing and symptom segmentation are done using a variety of new techniques.

In 1970, various image processing techniques were employed to tackle challenges connected to naked eye inspection in an agricultural field. In the agricultural industry, several existing procedures consider segmentation of distinct crop components such as fruits, stem and leaf, extracting and identifying different diseases and spots created by stress [6]. When a big number of training samples are not available to process, traditional image processing approaches function well. Some of the drawbacks related to plant disease detection are the fusion of background and object regions, similarity and shapes extracted during feature extraction. As a result, recent deep approaches effectively addressed the majority of the aforementioned issues.

Several conventional machine learning approaches have gained more practice in identifying and diagnosing plant lesions and are limited to image segmentation, feature extraction, and pattern recognition procedures [7]. It is impossible to extract features from a large amount of dataset using traditional techniques such as manual detection and machine learning. A deep learning model, on the other hand, retrieves the data abstraction from bottomless layers that are more useful and valuable than the machine learning techniques that cannot be applied on image dataset. The inclusion of redundant feature information is one of the drawbacks of employing deep architecture [8]. For classification, Convolutional Neural Network (CNN) [9] architecture incorporates some layers such as ReLu, Max-Pooling, Convolutional, Softmax and Fully Connected Layer [10]. A large amount of data, without any segmentation, is required to train a CNN model. Normally, raw data is fed into a CNN model as an input. VGGNet [11], ResNet, AlexNet, and Inception V3, EfficientNet are the most popular and advanced architectures in CNN [12]. PlantVillage is a typical training dataset that contains roughly 54,000 plant leaf picture datasets with different 36 classes, and the models are trained on it. It is always not possible to obtain a large amount of dataset for the training of models. The researchers employed the Transfer Learning (TL) method for this. They used this method to retrain the model on a specified dataset with the same parameters. When comparing traditional techniques to CNN models, transfer learning techniques are preferable due to the number of characteristics extracted. Due to its excellent generalization ability and robustness, the transfer learning approach with deep learning techniques excels in many areas, including the processing of signals, identification, and recognition of face data, road crack detection, and medical image analysis. Furthermore, these techniques have achieved promising output in the area of farming with disease detections, benefiting more smallholders and horticultural workers [13], in areas such as crop disease diagnosis [14], weed recognition, fine seed selection, pest detection, fruit counting, and land cover research, among others, which has contributed to image classification.

The novel contributions in this research work involves pre-processing of digital images and extracting their feature planes, followed by classification based on the type of leaf disease. Also, the use of different pre-processing techniques focuses our analysis on the leaf disease, as this element contains all of the information regarding the leaf illness. Color feature information has also been added as an image to determine if it may aid the classification process in correctly detecting disease utilizing Hybrid Convolutional Neural Network (Hy-CNN) transfer learning and more accurate feature reduction.

The content in rest of paper is described as follows: Section 2 discusses about related works; Section 3 is important as it describes the detail of dataset and classifies the stress of leaf using proposed Hy-CNN model. Section 4 discusses experimental result in terms of performance analysis. Section 5 discusses in terms of comparison with other models. Finally, Section 6 shows conclusion and future scope.

## 2. Related Work

An automatic model for detecting and classifying an unhealthy area of plants is discussed by Atila et al. [15]. The accuracy of the Efficient Net CNN-based state-of-the-art model was compared with different models in order to detect various diseases of a plant leaf. The accuracy achieved with this model is 96.18% as compared to different architectures. The united Convolutional Neural Network for the identification of disease in grape plant is mentioned by Ji et al. [16]. United Model’s representational potential is bolstered by high-level feature fusion, allowing it to outperform the competition in the grape leaf diseases identification mission. The F-CNN and S-CNN model [17] with full image and segmented images to classify and detect disease in plant leaves. When the trained CNN model is applied to a segmented image instead of to a complete image, the accuracy achieved is more than the full image. The 23-layered deep CNN model is compared it with all other different and machine learning models in terms of accuracy is discussed by Azimi et al. [18]. As compared with other features, the nitrogen stress features are easily classified using the proposed CNN model. Disease caused various loos both in the field of crop production and economy growth. Gadekallu et al. [19] introduce a hybrid PCA technique with optimized algorithm named whale optimization for feature extraction and evaluated the data in terms of accuracy and superiority. Rust and Cercospora are the primary two diseases that affect the quality and productivity of the coffee plant. The texture features for extraction using k-means and thresholding segmentation algorithm is done by Sinha et al. [20], and then the relation between infected part and healthy part is identified using texture analysis on the olive plant. Sorte et al. [21] suggested texture-based pattern recognition algorithm to detect leaf lesions on the coffee plant. The attributes (local binary attribute and statistical attribute) are calculated and compared with the CNN identification rate. The performance of deep learning models in terms of “learning rate”, “batch size”, “activation function type” and “regularization rate” using tensorflow application is calculated by Kallam et al. [22]. With these terms the author finds the number of hidden layers with test and training loss. The classification of Okra’s plant disease [23], depending on pod length, using different techniques. Different models are used to recognize wormholes, insects, and pests with AlexNet, GoogleNet, and ResNet50. The accuracy achieved using ResNet50 is better than other techniques. A CNN model with eight hidden layers that perform well than machine learning techniques is well explained by Franczyk et al. [24].

With some traditional techniques the detection process requires high cost, more human intervention and maintenance. With “Automatic and Intelligent Data Collector and Classifier” the scope for detecting and visualization of disease become easier and cost effective well explained by Kundu et al. [25]. Bacterial, fungus, algae, nematodes are some of the common diseases on plant leaves. The disease analysis expert is required for the detection of diseases in plant at early stage. Using color feature [26] techniques the feature vector extracts the common disease features and passes on the values to the proposed classifier for detection and classification of leaf disease. With some deep learning techniques and pre-processing the number of unbalanced images is balanced for training and testing by Oyewola et al. [27]. For color stretching the gamma correction and decorrelation is applied. The result depicts the better accuracy with balanced dataset. Using color distribution the trainable images are modified and recognize the diseases using color space transformation technique. The image histogram transformation technique is employed by Abayomi-Alli et al. [28]. A model for identification of a number of lesions that cause damage to crops which led to a shortage of cultivation is discussed by Basavaiah et al. [29]. Using plant village datasets of different classes are classified using different techniques and achieve maximum accuracy of 98%. One of the most significant operations in precision agriculture is image-based fungal disease prediction for detecting the occurrence and quantifying the severity of variability in crops. Some work with machine learning models for comparative analysis with SVM is discussed by Abdu et al. [30]. Both models were deployed on a dataset of large-scale horticulture leaf lesion pictures using conventional surroundings and considering the critical elements of architecture, processing capacity, and training data. Convolutional Networks are a type of Neural Network that has shown to be particularly good in image recognition and classification. Different techniques are covered under CNN Network for the classification of disease using images of all fields such as medical images [31], hand gesture images, disease images, and diabetic images [32]. Techniques such as VGG16, VGG19, ResNet, Inception, MobileNet, and EfficientNet are pre-defined models for the better classification of the segmented part. Convolutional Neural Networks offers better accuracy result as given in TTA algorithm with feature extraction and classification technique.

Table 1 obtained by various authors using pre-trained deep learning models, which were obtained according to the dataset each study employed, in an arbitrary order.

With different recent deep learning technologies, a comparison is also being performed. These studies concentrated on the issue of overfitting and computational time. They used a number of approaches to accomplish this, including selecting crucial traits for accurate classification. Furthermore, instead of using many classifiers for a fair comparison, they focused on one classifier for features classification. As per various discussed works that there exist some gaps for research works, i.e., the number of classes and optimization tradeoffs, types of diseases classified, epochs optimization, better accuracy, etc., that need to be addressed in the use of architectures in horticulture plant leaf lesion detection. Majorly effectiveness of classes and accuracy of deep learning architectures offer the efficiency of the classifications. In this work, to enhance the amount of training image samples, high correlation function are used to accomplish the data. Then, for improving pixel intensity of an image, normalizing technique was done.

## 3. Materials and Methods

For proper pest management and fertilizer application, early detection of diseases in grape leaves is required as per gaps identified by the literature covered. Farmers may produce low-profit yields despite their hard work if these biotic stresses are not identified promptly. Several image processing algorithms have been developed to detect lesions, alerting farmers and allowing for early diagnosis.

### 3.1. Data Materials

**Figure 1:**
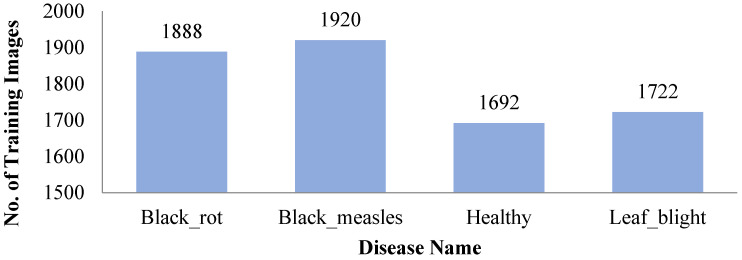
Number of Training Dataset.

The diseases such as powdery mildew, downy mildew, rust, black rot, scab, etc., [16] are found on grape leaves. The dataset is collected from PlantVillage [33] which is composed of healthy images (2115 images), including diseased images of black rot (caused by ascomycetous fungus; 2360 images), black measles (caused by phaeomoniella aleophilum fungus; 2400 images), and leaf blight (caused by pseudocercospora Vitis fungus; 2152 images) as shown in Figure 1 below.

All the images are resized to 224 × 224 × 3. A total of 9027 images of the grapes crop from are split into a ratio of 80:20 for validation and testing as displayed in Table 2 with symptoms. This study calculates the accuracy or performance of several approaches.

**Figure 2:**
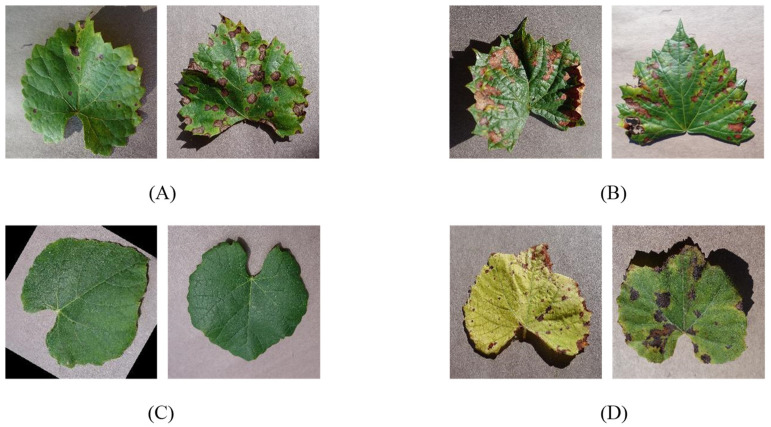
Few samples grape leaf image dataset. (**A**) Black rot leaf image; (**B**) Black_measles leaf image; (**C**) Healthy leaf image; (**D**) Leaf_blight leaf image.

Only the leaf part is considered to identify lesions in grape leaves because the grape flower and fruit are short-lived, while the leaf is present throughout the year. Furthermore, the stem of a grape will rarely present disease symptoms promptly, while the leaf’s shape, texture, and color are all affected by the state of the plant, typically provide more detail. There are few sample images collected from plant village of grape disease leaf dataset are shown in Figure 2. 

### 3.2. Proposed Hy-CNN Model

**Figure 3:**
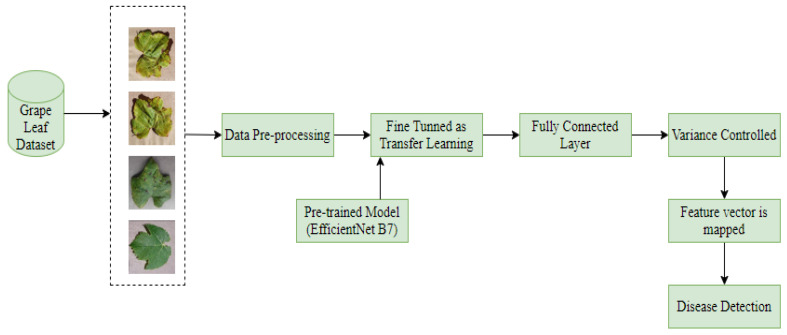
Hy-CNN model block diagram for grape leaf disease detection and classification.

In this part, for the detection and classification of grape leaf disease using “Deep Transfer Learning” a proposed model approach is consisting of four different steps:—data pre-processing (Resizing and normalizing the dataset), training of deep CNN model with transfer learning, feature reduction and classification technique for classify the disease data. The Hy-CNN model used block diagram is composed of different steps, as given in Figure 3.

#### 3.2.1. Image Pre-Processing

**Figure 4:**
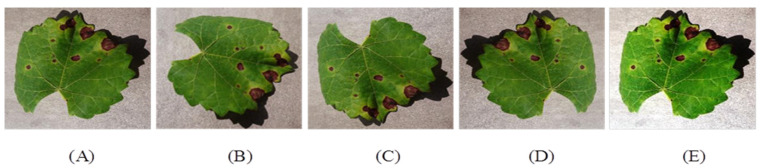
Data Pre-processing (**A**) Original image, (**B**) 90° rotated image, (**C**) Vertically flipped image, (**D**) Horizontally flipped image, (**E**) Intensified image.

According to the literature, preprocessing of images is the most significant operation that must be performed to obtain an appropriate data with no undesired distortions and highlight the picture properties that will be relevant for later processing [34]. The images in the dataset are downsized to 224 × 224 × 3 resolutions to speed up the training process by applying a dataset of homogeneous images. It changes the representation of an image, i.e., its color, shape, texture, or removes noise [35]. Another key issue is overfitting, which occurs when a large number of pictures produce random noise. For the preventative measures against overfitting the correct range of training and testing dataset is collected with correct dimension of data. As the data have different intensity values and scale that are used at the time of training, all photos are then normalized to the same scale. Image normalization is a technique for narrowing the range of pixel intensity. Equation (1) shows the general form of normalization.

(1) $$F_{norm}=\frac{\left(F-F_{min}\right)}{\left(F_{max}-F_{min}\right)}$$

where, *F* denotes the normalization value, *F_min_* minimum pixel value, *F_max_* maximum pixel intensity value w.r.t an image. For increasing the size of dataset, different data augmentation technique is followed such as rotation, flipping and image brightness. Figure 4 shows different image samples after augmentation.

#### 3.2.2. EfficientNet

Since 2012, as the utilization of the models increases for the training of ImageNet dataset become complicated, success has increased, although many are ineffective in terms of compute burden. The EfficientNet model can be regarded a set of CNN models because it achieves an accuracy rate of 84.4 percent with 66 M parameter in the classification task of ImageNet dataset, making it one of the state-of-the-art models [36]. The EfficientNet model consists of 8 different models ranging from B0 to B7, the number of estimated parameters does not rise significantly as the number of models increases, while accuracy increases dramatically. The EfficientNet utilizes a new activation function called Leaky ReLu activation function in place of Rectifier Linear Unit (ReLu). EfficientNet, unlike other state-of-the-art models, produces more efficient results by scaling width, resolution and depth uniformly when the model is scaled down. The initial stage in the compound scaling strategy under a fixed resource limitation is to look for a grid to determine the relationship between the different scaling dimensions of the baseline network [37].

The key building component introduced by MobileNet V2 i.e MBConv bottleneck was used by EfficientNet, but is employed significantly more than MobileNetV2 because to the increased “Floating point operations per second” (FLOPS) budget. Direct connections are used between bottlenecks with considerably fewer channels than expansion layers in MBConv because blocks are made up of a layer that expands and then compresses the channels [35]. The calculation is reduced by k_2_ factor as the as the layers design get separate, here k is the kernel size, which represents the width and height of the 2D convolution window.

Mathematically, EfficientNet is defined in (Equation (2)) as:

(2) $$P=\sum_{x=1,2,\ldots n}M_{x}^{T_{x}}\;\left(Y_{\left(A_{x},\;B_{x},\;D_{x}\right)}\right)$$

where, $M_{x}$ represents the layer mean and repeats $T_{x\;}\;$ times in variance of *x*. $\left(A_{x},\;B_{x},\;D_{x}\right)$ represents the shape input in tensor of *Y* w.r.t the layer *x*. The inputs of the images change from 256×256×3 to 224×224×3. For increasing the model accuracy, the layers must scale with a proportional ratio optimized with the given formula:

(3) $$max_{x,y,z}\;\;\;\;\;\;Acc\left(P\left(x,y,z\right)\right)$$

(4) $$P\left(x,y,z\right)=\sum_{s=1,2,\ldots }M_{s}^{L_{s}}\;(Y_{\left(z.A_{s},\;z.B_{s},\;y.D_{s}\right)})$$

Memory(P) <= destinated_memory

FLOPS(P) <= destinated_flops

**Figure 5:**
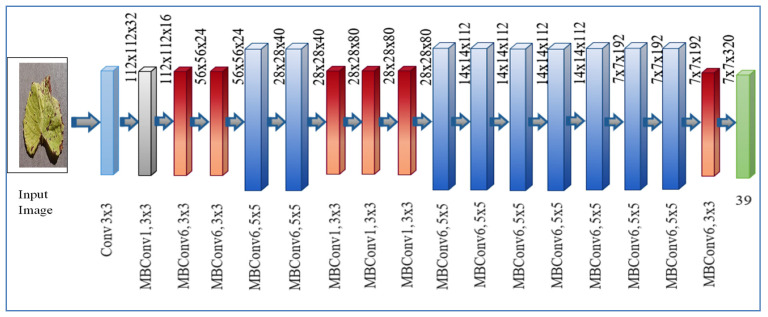
Systematic diagram of EfficientNet B7 architecture for leaf disease detection.

The height, breadth, resolution is represented with *x, y, z* in Equation (3). A number of layers used in model with details of parameters are shown in Equation (4) and Table 3. A systematic diagram of EfficientNet B7 is represented in Figure 5.

Some of the few abbreviations used in paper are mention in Table 4, which further used to preprocess images, Segmentation, Feature Extraction, and Classification.

#### 3.2.3. Training of Model Based on Transfer Learning

**Figure 6:**
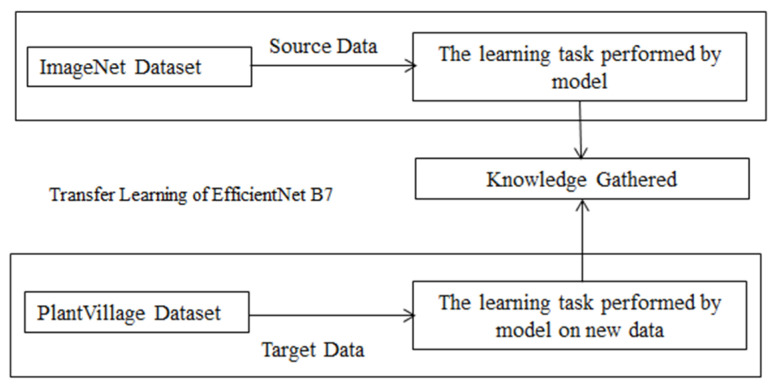
Re-training of proposed model.

Deep learning, which is more modern, has had a major impact in a variety of fields. In the agricultural arena, gathering a large quantity of data for training of model that is new from start is difficult. The training data determines how well a system performs. With transfer learning the data get independent with this approach. It is simple to train a model from an insufficient model using transfer learning [38]. In this study, the EfficientNet B7 CNN model is used for training on the PlantVillage dataset. For the prediction of grape leaf disease this transfer learning model is used. The model is trained on raw data with resized image from 256 × 256 to 224 × 224. The model once trained on this raw data get knowledge of detecting the disease on plant leaf. The trained model then applies on grape leaf dataset for the detection and identification of leaf disease. The working of re-train model based on transfer learning is shown in Figure 6.

#### 3.2.4. Feature Extraction

The FC layer is used to activate features after retraining a CNN model. The Leaky ReLu activation function is used to extract features from this layer. The extracted vector has a one-dimensional length of Nx1000 and a total length of. However, it has been discovered through random trials that all retrieved traits are not significant for last classification. Furthermore, the presence of those non-essential elements reduces the system’s efficiency [39]. The ReLU activation function has been improved with the Leaky ReLU function. For all input values less than zero, the gradient of the ReLU activation function is 0, deactivating the neurons in that region and perhaps creating the dying ReLU problem.

The term “leaky ReLU” was coined to describe a solution to this issue. We specify the ReLU activation function as an extremely small linear component of x instead of declaring it as 0 for negative values of inputs(x). This activation function’s formula is as follows in Equation (5):

(5) T(i)=max(0.01×i,i)

If the input is positive, this method returns x, but if the input is negative, it returns a very little number, 0.01 times x. As a result, it also outputs negative values. The gradient of the left side of the graph now has a non-zero value as a result of this tiny change. As a result, there would be no more dead neurons in that area.

#### 3.2.5. Feature Reduction

Feature relevance scores are important in predictive modelling projects because they provide insight into the data, insight into the model, and the foundation for dimensionality reduction and feature selection, which can improve the efficiency and efficacy of a predictive model on the problem [40]. It cuts down on the amount of time and storage space needed. It aids in the removal of multi-collinearity, which improves the interpretation of the machine learning model’s parameters. When data is reduced to very low dimensions, such as 2D or 3D, it becomes easier to visualize.

It avoids the dimensionality curse. Features are vital in pattern recognition since they help to describe the item. The quality of features is critical for accurate classification. Because the features that are extracted from the source image data are insufficient for the recognition of different variety of reasons, including feature redundancy and noisy features, a strategy for feature reduction or optimization is necessary. For feature reduction a new approach name Logistic Regression is used in this work. Variance is commonly used to determine how each pixel differs from its neighbors and to classify pixels into distinct areas. Furthermore, it is beneficial to compare the differences between two virtually identical photos [41,42]. Logistic regression is used to predict the output of a categorical dependent variable. As a result, the outcome must be either discrete or categorical. It can be 0 or 1, Yes or No, true or false, and so on, but rather than giving exact values like 0 and 1, it gives probabilistic values that are somewhere in between.

Logistic Regression is very similar to Linear Regression. The Linear Regression is employed for the regression problems, while for classification difficulties, Logistic Regression is used. Mathematical step for getting the logistic regression is given (Equation (6)).

(6) $$\log\frac{m}{1-m}=a_{0}+a_{1}b_{1}+a_{2}b_{2}+a_{3}b_{3}+\ldots +a_{n}b_{n}$$

The Algorithm 1 describes the complete process of Hybrid Convolutional Neural Network (Hy-CNN) model with feature extractor and shows the result in 0 or 1.
**Algorithm 1:** Training and Testing of Hy-CNN.**Input:** Grape image data loader batch **Output:** Trained Model **Processing Steps:** 1. If a set is training data set, then follow steps 2 to 5. 2.  resize to dimension (256 × 256) 3.  pre-processing to resize the image (224 × 224) 4.  normalize pixel values [0, 1] 5.  standardize pixel values to (256 × 256) 6. If a set is (224 × 224), then follows steps 7 to 9 7.  normalize pixel value [0, 1] 8.  standardize pixel values to (256 × 256) 9.  augment the data with different augmentation techniques 10. Model training with MODEL = EfficientNet B7 11. for a model in MODEL use steps 12 to 13 12.  fined tuned with transfer learning 13.  use Leaky ReLu activation function 14.  for epoch = 30, 50, 70, 100. 15.      Set learning rate 0.001 use steps 16 to 17 16.  for image in data loader batch: 17.      update model parameter 18.  end of for loop of step 16 19.  if training accuracy does not improve for 9 epochs, follow steps 20 to 21
20.     
$\mathrm{then}:\;j_{s}=j_{s}\times 0.1$
21.      unfreeze feature layers
22.     
$j_{s}=1e^{-4}$
23.  end of for loop of step 14 24. end of for loop of step 11 25. for epochs = 30, 50, 70, 100 use steps 26 to 29 26.  for a testing image in data loader batch: update model parameter 27.  end of for loop of step 26 28.  if testing accuracy does not improve up to 7 layers
29.     
$\mathrm{then}:\;j_{s}=j_{s}\times 0.1$
30. end of for loop of step 25

Training and Testing Algorithm (TTA) accepts as input of Grape image data loader batch (Bh) 256 × 256 × 3 and after preprocessing resize the image 224 × 224 × 3. Now normalize the pixel value range, which varies from 0 to 1 with the same standard pixel value.

For the training data, freeze the feature layers $j_{s}=1e^{-3}$ with different epochs and a learning rate of 0.001. For increasing the training accuracy of the data, the images are uploaded with more parameters. If training accuracy does not improve with the given parameters, unfreeze the features where the layers$\;j_{s}=1e^{-4}$. If the testing data accuracy does not improve up to 7 layers, increase the layers $j_{s}=j_{s}\times 0.1$ and achieve the best performance based on the CNN model.

### 3.3. Statistical Analysis

A dataset of Grape leaf disease photos was employed in the suggested EfficientNet B7 model, which contributed to statistical analysis parameters. With a size of 224 × 224, the entire dataset was statistically significant at the 0.05 level. It provides significant evidence against the null hypothesis, as the null hypothesis has a less than 5% chance of being right using one-way ANOVA method using SPSS-2021, IBM, USA. The significant difference was determined when *p <* 0.05. The parameter accuracy, efficiency and precision values are evaluated statistically for their statistical parameters like mean and standard deviations. The parameters accuracy, efficiency and precision are found to be statistically different at *p ≤* 0.05. This shows the effect of our proposed model on these parameters. In the data analysis, the various four classes of dataset particular exposure for general impairment have been understood. After 92 epochs, the most discriminant features are identified with the maximum constant accuracy of 98.7% utilizing state-of-the-art classifiers using the suggested EfficientNet B7 model. Based on the confusion matrix of dataset variance, this research looked at Precision, Recall, and F1-Score. As a result, choose the confusion matrix to limit the danger of job status and outcome.

## 4. Results

**Figure 7:**
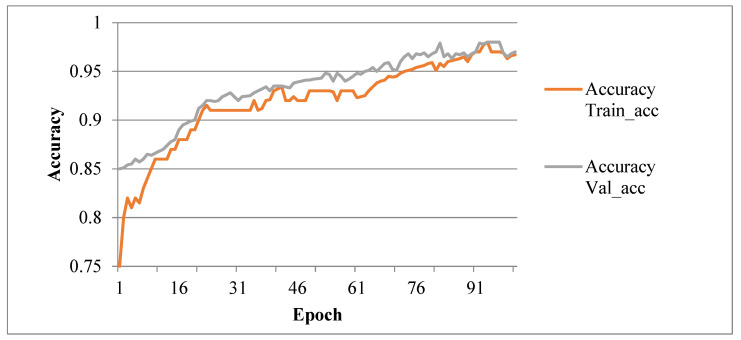
Accuracy Graph of Hybrid Convolutional Neural Network (Hy-CNN).

In this work, CNN based Efficient B7 model is tested and validated to detect lesions on plant leaves. Python-based algorithms are used for image preprocessing with Anaconda3 (Python 3.6), Keras-GPU library, and OpenCV-python3 library. These are used for data augmentation and CNN, respectively. For this experimental setup, GPU (Graphics Processing Unit) with 12 GB RAM and 68 GB Hard Disk is used to accelerate Deep CNN training and testing. The Grape leaf dataset is taken from the plant village for training and testing to evaluate the Hy-CNN approach’s performance. The 30, 50, 70, 100 epochs value are used for training Hy-CNN model. The accuracy obtained from each epoch is plotted in the graphs shown in Figure 7. The model’s accuracy is shown in Table 5 per each epoch.

### Performance Evaluation

In this work, there are few different constraints such as “precision value”, “recall”, and “F1 score” are given below in Equation (7), Equation (8), and Equation (9) respectively. Precision is an association of different set of the data samples that are estimated to each other in term of disease detection in grape leaf. The association of four different classes such as “Black_rot”, “Healthy”, “Black_measles” and Leaf_blight” is 0.964.

(7) $$\mathrm{Pre}=\frac{\mathrm{TP}}{\mathrm{TP}+\mathrm{FP}}$$

where TP: True Positive, FP: False Positive.

The recall takes the responsibility of relevant cases of different classes of data association in the disease detection of grape leaf dataset in 2 different cases “True Positive”, “False Negative”.

(8) $$\mathrm{Rec}=\frac{\mathrm{TP}}{\mathrm{TP}+\mathrm{FN}}.$$

where TP: True Positive, FN: False Negative.

F1-score is evaluating the two-way classification (Precision and Recall) of the given dataset.

(9) $$F1\mathrm{Src}=\frac{2\times \mathrm{Pre}\times \mathrm{Rec}}{\mathrm{Pre}+\mathrm{Rec}}$$

There are four classes of grape leaf such as Leaf blight, Healthy with Black measles, and Black rot. The “True Positive (TP)”, “True Negative (TN)”, “False Negative (FN)”, and “False Positive (FP)” are some of the duration terms of this context; true positive represents a healthy leaf. In case of true negative the leaves are incorrectly identified as healthy leaf. The false positive represents the correct identification of diseased leaf images. The correct identification is identified incorrectly as plants leaf in a False Negative [43,44].

## 5. Discussion

There are two different Convolutional network models for image classification such as “Image Classification” (IC) and “Object Detection” (OD) is discussed in Table 6. The image classification and detection are the two basic terms for image recognition.

Based upon effective results shown in Table 6, the Hybrid Convolutional Neural Network (Hy-CNN) model shows the highest accuracy of 98.7% for identification of leaf disease after 92 epochs with largest F1 score value as compared with others [45]. Comparable effects, specifically constrained recall, had been found for EfficientNet B7 trained to detect leaves inside the grapes crop. The researchers analyzed the uniqueness of Convolutional Neural Network (CNN) model for identification of disease with different epochs. The comparison of Hy-CNN model with some deep learning models is shown in Table 7. Different varieties of deep learning models are used for the detection, recognition and characterization of lesion detection in plant leaf. Some authors implement different Convolutional Neural Network (CNN) models such as EfficientNet—CNN [13], united deep learning model [15], F-CNN & S-CNN [17] on different plant leaf images. Some suggested proposed models with less accuracy as 92.01% [18], some with hybrid analysis model [19] with an accuracy of 95.1% on plant leaf. For more clear classification some texture image analysis [21] is implemented on coffee leaf images.

With some deep learning models, depending upon the layers of model some show more better rest for the classification of okra plant leaf [23] and coffee leaf [30]. The Hybrid Convolutional Neural Network (Hy-CNN) transfer learning model shows efficient result for the detection of grape leaf lesions using feature extractor model. Efficient-Net B7 is a convolutional neural network architecture and compound scaling method that uniformly scales all dimensions of depth/width/resolution using a compound coefficient but different input sizes in the same method create an issue. It has also linearly increased dropout ratio such as from 0.2 for EfficientNet-B0 to 0.5 for B7. EfficientNet models use 37 Billion FLOPS by an order of magnitude (up to 8.4× parameter reductions and up to 16× FLOPS reduction) for this it uses e GPU which has the time-consuming process.

## 6. Conclusions

This paper presents a new deep transfer learning-based model for detecting grape plant leaf lesions. Using FC layer the features are extracted, and then, using proposed variance technique extraneous features are eliminated from the feature extractor vector. The resulting characteristics are classified using classification algorithms with 98.7% classification accuracy. Based on the findings, it is summarized that with the transfer learning model the training become more mature as compare to training the model from the ground up. Furthermore, the next step of reducing characteristics will improve the classification accuracy. For image processing and classification, algorithms have gained popularity. Dataset is cleaned by reducing the resolution to downgrades. Considering the following optimization parameters such as dropout ratio, FLOPS size by an order of magnitude, processing speed, accuracy, efficiency and precision metric, the Hybrid Convolutional Neural Network (Hy-CNN) is superior to other CNN architectures. Also conclude that the fitness function become the desirable feature selection function for calculating the accuracy. The dataset is divided into 80:20 strategy based on the outcomes. The fundamental drawback of this work is data availability, which is mitigated in part by incorporating a data augmentation stage. Similarly, selecting the most relevant features is critical; otherwise, the total classification accuracy may suffer. It did well in classifying grape leaf diseases, but the guaranteed/universal strategy to selecting the most valuable traits is still absent. The plant leaf disease dataset can be expanded by boosting plants diversity and the number of classification classes. Then another future direction is fine-tuning the DL model based on its epochs to suit the need of datasets and their classes to categorize. Also used the morphological properties such as texture (color, shape and size), and spectral for the detection of leaf disease. This will aid in developing models that can make more accurate predictions for more diverse diseases of plants in the future.

## Figures and Tables

**Table 1:** The result of the state-of-art models.

Paper (Ref.)	Dataset Used	Precision	Recall	Accuracy
[16]	1619	80.20%	95.2%	98.28%
[17]	1350	85.2%	89.2%	98.35%
[18]	16,579	90.1%	91.00%	92.01%
[20]	1200	87.0%	85.2%	90.21%
[21]	1507	89.12%	82.5%	97.00%

**Table 2:** Description of Training, Testing Data with symptoms.

Type	Training Images	Testing Images	Class	Symptoms
Black_rot	1888	472	1	Black spherical fruiting and brown circular lesions.
Black_measles	1920	480	2	Dark brown-black vascular streaking.
Healthy	1692	423	3	Green in color.
Leaf_blight	1722	430	4	Soaked spots on the lower surface of leaves.

**Table 3:** Model layer and parameter categorization.

Level	Layers	Resolution	Number of Channels	Number of Layers
1	Convo_3 × 3	224 × 224	32	1
2	MBConvo1_3 × 3	112 × 112	16	1
3	MBConvo6_3 × 3	112 × 112	24	2
4	MBConvo6_5 × 5	56 × 56	40	2
5	MBConvo6_3 × 3	28 × 28	80	3
6	MBConvo6_5 × 5	14 × 14	112	3
7	MBConvo6_5 × 5	14 × 14	192	4
8	MBConvo6_3 × 3	7 × 7	320	1
9	Convo_1 × 1 with Pooling and FC layer	7 × 7	1280	1

**Table 4:** Abbreviations used in this work.

Notations	Meaning
Rec	“Recall Value”
Pre	“Precision Value”
F1Src	“F1 Score”

**Table 5:** Accuracy table of Training and Testing with models.

Model Name	Number of Epochs	Training Accuracy for Each Epochs	Testing Accuracy for Each Epochs
Hybrid Convolutional Neural Network (Hy-CNN)	30	96.2%	95.1%
	50	95.4%	96.1%
	70	97%	96.5%
	100	98.7%	97%

**Table 6:** Parametric data calculation of precision, F1-Score and recall.

CNN Network Model	Network	Precision Value (Maximum)	Recall	F1-Score
Hybrid Convolutional Neural Network (Hy-CNN)	IC	0.95	0.22	0.94
	IC	0.95	0.21	0.94
	OD	0.97	1	0.96
	OD	0.98	0.99	0.97

**Table 7:** Comparison of Hy-CNN model with existing deep learning models.

Ref. No.	Method	Accuracy	Plant Name
[15]	EfficientNet—CNN	96.18%	Plant Leaf
[16]	United Model	98.2%	Grape Leaf
[17]	F-CNN & S-CNN	98.3%	Tomato Leaf
[18]	Proposed FCNN & SCNN	92.01%	Crop Leaf
[19]	Hybrid Principal Component Analysis	95.1%	Plant Leaf
[20]	Hybris PCA & Optimization Algorithm	90.2%	Apple Leaf
[21]	Texture and AlexNet	97.0%	Coffee Leaf
[23]	Deep Learning Models	98%	Okra Leaf
[33]	Decision tree & Random Forest	90% & 94%	Tomato Laef
[34]	Deep CNN	98%	Coffee Leaf
Hybrid Convolutional Neural Network (Hy-CNN)	Deep Transfer EfficientNet B7 model	98.7%	Grape Leaf

## Data Availability

Data is available in public access for research purpose at: Hughes, D. P.; Salathe, M. An open access repository of images on plant health to enable the development of mobile disease diagnostics. arXiv preprint arXiv:1511.08060 2015.
